# Constructing Relative Effect Priors for Research Prioritization and Trial Design: A Meta-epidemiological Analysis

**DOI:** 10.1177/0272989X231165985

**Published:** 2023-04-14

**Authors:** David Glynn, Georgios Nikolaidis, Dina Jankovic, Nicky J. Welton

**Affiliations:** Centre for Health Economics, University of York, UK; IQVIA, London, UK; Centre for Health Economics, University of York, UK; Bristol Medical School (PHS), University of Bristol, UK

**Keywords:** meta-epidemiology, bayesian methods, value of information, research prioritisation, hierarchical models

## Abstract

**Background:**

Bayesian methods have potential for efficient design of randomized clinical trials (RCTs) by incorporating existing evidence. Furthermore, value of information (VOI) methods estimate the value of reducing decision uncertainty, aiding transparent research prioritization. These methods require a prior distribution describing current uncertainty in key parameters, such as relative treatment effect (RTE). However, at the time of designing and commissioning research, there may be no data to base the prior on. The aim of this article is to present methods to construct priors for RTEs based on a collection of previous RCTs.

**Methods:**

We developed 2 Bayesian hierarchical models that captured variability in RTE between studies within disease area accounting for study characteristics. We illustrate the methods using a data set of 743 published RCTs across 9 disease areas to obtain predictive distributions for RTEs for a range of disease areas. We illustrate how the priors from such an analysis can be used in a VOI analysis for an RCT in bladder cancer and compare the results with those using an uninformative prior.

**Results:**

For most disease areas, the predicted RTE favored new interventions over comparators. The predicted effects and uncertainty differed across the 9 disease areas. VOI analysis showed that the expected value of research is much lower with our empirically derived prior compared with an uninformative prior.

**Conclusions:**

This study demonstrates a novel approach to generating informative priors that can be used to aid research prioritization and trial design. The methods can also be used to combine RCT evidence with expert opinion. Further work is needed to create a rich database of RCT evidence that can be used to form off-the-shelf priors.

**Highlights:**

- Bayesian methods have potential to aid the efficient design of randomized clinical trials (RCTs) by incorporating existing evidence. Value-of-information (VOI) methods can be used to aid research prioritization by calculating the value of current decision uncertainty.
- These methods require a distribution describing current uncertainty in key parameters, that is, “prior distributions.”
- This article demonstrates a methodology to estimate prior distributions for relative treatment effects (odds and hazard ratios) estimated from a collection of previous RCTs.
- These results may be combined with expert elicitation to facilitate 1) value-of-information methods to prioritize research or 2) Bayesian methods for research design.

Empirical research provides the scientific foundation for modern medicine and must be carefully designed so that it yields valid results. Transparent research prioritization is also necessary, as budgets to fund research are limited.

In recent decades, Bayesian methods have been developed to aid study design and research prioritization as well as overcome limitations associated with frequentist approaches.^1,2^ Value-of-information (VOI) methods have been developed in the economic evaluation literature also to aid study design and research prioritization.^3,4^ VOI methods calculate the value of reducing or eliminating decision uncertainty in a particular clinical decision. As a starting point, VOI methods require a decision model that incorporates the best available evidence (with uncertainty) into a probabilistic mathematical model. This model can then be used to predict outcomes with different treatment options in addition to the uncertainty in these predictions. VOI metrics are based on Bayesian decision theory and are calculated by estimating the expected consequences of making an incorrect decision with current evidence.^5,6^

Bayesian methods typically require probability distributions, which represent beliefs on the parameters of interest prior to collecting further evidence. These distributions are referred to as priors. In data analysis, “noninformative” (or “flat” or “vague”) priors can be adequate to implement Bayesian methods.^4^ Noninformative priors will rarely be accurate representations of beliefs in clinical contexts because they ascribe probability across an arbitrarily large range of parameter values. In data analysis, this will be inconsequential if there are sufficient data to dominate the posterior distribution. However, in the context of VOI methods, priors must reasonably represent beliefs in order to generate meaningful results.

The key parameter that clinical research is designed and commissioned to inform is the relative treatment effect (RTE), such as odds ratios, hazard ratios, or mean difference. Randomized controlled trials (RCT) are required to gather reliable information on RTEs as they allow for the comparison of treatments while controlling for selection effects and confounding more generally. In the VOI literature, previous RCTs answering the clinical question of interest are often used to inform priors for the RTE.^3,7^ In this approach, a distribution on an appropriate scale (log, logit, or natural) is constructed based on the published confidence interval or standard error to represent the uncertainty around the RTE.^8^ These metrics may come from a previous study or a meta-analysis or from expert elicitation.

As discussed above, to use VOI for research prioritization requires a decision model. Ideally, all of the inputs to this model would be based on a large number of relevant high-quality studies. However, it has been found in practice that at the time of research prioritization decisions, there are often few if any previous studies that can be meta-analyzed to inform an appropriate RTE prior.^9–11^ This should not be surprising, as research is often required because there is a lack of good-quality research addressing a specific question. Therefore, if VOI is to be used routinely by research prioritization bodies to make decisions, it will be necessary to make some judgment about RTE priors. Because of the decision context, this prior will necessarily be imperfect. Therefore, a model incorporating such a prior may be appropriate for research prioritization decisions but not appropriate for reimbursement decisions until further evidence has been collected and used to inform the RTE parameter.

Noninformative priors could be used for RTEs; however, as discussed above, these priors are arbitrary and unlikely to represent beliefs about the plausible distribution of RTE values. More sophisticated methods are available for this task. These methods are described in more detail in Appendix 1. Briefly, these include 1) structured expert elicitation, which is a process used to formally capture the beliefs of individuals identified as experts in a particular area^12^; 2) sharing information from indirectly related evidence, which is the process of combining evidence relating both directly (on the exact population and treatments of interest) and indirectly (on related populations or treatments) to a research question to predict outcomes in the context of interest^13^ and; 3) meta-epidemiological analysis.

Murad and Wang^14^ stated that meta-epidemiological studies “adopt a systematic review or meta-analysis approach to examine the impact of certain characteristics of clinical studies on the observed effect and provide empirical evidence for hypothesized associations. The unit of analysis in meta-epidemiological studies is a study, not a patient.” A meta-epidemiological approach can be used to from RTE priors by exploring results from RCTs across a range of disease areas to obtain a realistic distribution for the likely values that RTEs may take in the context of interest. The intuition for this approach is that it is possible to learn about the plausible range of a RTE in a new trial by principled analysis of RTE in previous trials in similar disease areas. It would be unlikely for a new RTE to be much bigger than that seen in previous RCTs, unless there was a substantial advance in the mechanism of action of the new treatment.

## Aim of This Article

RTE priors are necessary for VOI to be used in applied research prioritization. The literature on constructing these priors using expert elicitation is well established, and work is emerging on indirect information sharing, but there is limited research on the meta-epidemiology approach. The aim of this article is to provide a novel methodological framework to construct priors for RTEs based on meta-epidemiological analysis. We illustrate the methods by analyzing the database of RCTs used in Djulbegovic et al.,^15^ to construct a set of priors for RTEs.

We demonstrate how such priors can be used to compute VOI for a case study of high-grade non–muscle-invasive bladder cancer and compare our results with the VOI estimated when using noninformative priors. The “Discussion” section outlines a number of ways priors may be used and combined with other methods. We also discuss the data sets required to produce more appropriate prior distributions.

## Methods

This section outlines a meta-epidemiological approach to estimating predictive distributions that can be used as informative priors for the RTE of a future trial.

Deriving a reasonable prior for a new study requires 2 steps. First, the observed results in a representative data set of historical RCTs were modeled using a hierarchical model to estimate the distribution of RTEs based on the existing evidence. In the second step, the RTE in the new RCT was predicted from the hierarchical model fitted to the historical RCTs. This assumed that the true effect in the new RCT was exchangeable with those seen in the historical data set after accounting for any covariates included in the model. This assumption is reasonable if covariates that may modify the RTE have been accounted for and we do not have further reason to expect that the true effect in the new trial will be systematically different from the true effects observed in the set of past trials.^4^ If the exchangeability assumption holds, then the predictive distribution from the hierarchical model can be used as an informative prior for the true effect in a new study.^4^

### Hierarchical Statistical Models

We present 2 hierarchical statistical models. Model 1 allowed for a separate mean effect for each disease area and disease area–specific between-study variances. Information sharing was permitted between both means and between-study variances. Model 2 extended this to take account of covariate effects for comparison type (i.e., active v. active or active v. inactive treatment).

#### Model 1: Hierarchical model for disease-specific means and between-studies variances

We assumed that each study i reports a summary treatment effect estimate $y_{i}$ and a standard error $se_{i}$ . Following Djulbegovic et al.,^15^ we pooled data for log odds ratio and log hazard ratio summary treatment effects. We assumed that the study samples are sufficiently large so that the likelihood can be considered approximately normal, such that

$$y_{i}\;\sim \;N\;\left(\theta_{i},{\mathrm{se}}_{i}^{2}\right)$$

where $\theta_{i}$ denotes the study-specific true effects. Since our data set comprises studies in several different disease areas and the distribution of true relative effects of RCTs may differ across disease areas, we allowed for $\theta_{i}$ to accommodate the hierarchical structure of our data such that

$$\theta_{i}\sim N\;\left(\mu_{A},\;\tau_{A}^{2}\right)$$

$$\mu_{A}\;\sim \;N\;(M,\;\eta^{2})$$

$$\tau_{A}^{2\;}\;\sim \;\pi \;(\kappa ,\;\nu )$$

where A indexes the disease area and $\mu_{A}$ and $\tau_{A}^{2}$ denote the disease area–specific true effect means and between studies variances, respectively. We assumed that the disease area–specific means are exchangeable and come from a normal distribution of disease area mean effects with an overall mean across disease areas M and a between disease areas variance $\eta^{2}$ . This model therefore borrowed strength across disease areas by shrinking the estimates toward the overall mean M .

Similarly, we also enabled information sharing across disease areas on the heterogeneity parameters by letting $\;\tau_{A}^{2\;}$ follow a distribution π(.) parametrized by κ and ν . Three alternative distributions π(.) were explored, namely, a log-normal (model 1a), a Gamma (model 1b), and a half-normal (model 1c).

Noninformative priors were assigned to $M\;\sim \;N(0,\;1000^{2}),\;\eta \;\sim \;Unif(0,5)$ , and the parameters of π(.) . For the log-normal, $mean\;\sim \;N\;(0,\;1000^{2})$ and sd~Unif(0,5) ; for the Gamma, shape~Unif(0,50) and scale~Unif(0,50) ; and for the half-normal, $mean\;\sim \;\mathrm{Half}-\mathrm{Normal}\;(0,\;1000^{2})$ , truncated at the 0 to 100 range, and sd~Unif(0,5) .

Tailored predictive distributions for the disease areas included in our data set were derived as shown:

$$\theta_{pred}\sim N\;\left(\mu_{A*},\;\tau_{A^{*}}^{2}\right)$$

which captured the uncertainty in disease-specific mean true effect and disease-specific between-trials variance. To model a disease area that was not included in our data set, 3 levels of prediction were required: 2 levels to predict the parameters (mean and variance) of the predictive distribution of the true effects and a final level for the predictive distribution of the true effects, so that

$$\mu_{pred}\;\sim \;N\;(M,\;\eta^{2})$$

$$\tau_{pred}^{2\;}\;\sim \;\pi \;(\kappa ,\;\nu )$$

$$\theta_{pred}\sim N\;\left(\mu_{pred},\;\tau_{pred}^{2}\right)$$

#### Model 2: Hierarchical model incorporating covariate effects

Here, we extended model 1 to include a covariate that captures whether an RCT compares 2 active treatments or an active with an inactive treatment so that

$$\theta_{i}\sim N\;\left(\mu_{A}\;+\;\beta_{A}X,\;\tau_{A}^{2}\right)$$

$$\mu_{A}\;\sim \;N\;(M,\;\eta^{2})$$

$$\tau_{A}^{2\;}\;\sim \;\pi \;(\kappa ,\;\nu )$$

$$\beta_{A}\;=\;B$$

where X is a binary covariate that takes the value of 0 when an active treatment is compared with an inactive one and the value of 1 when an active treatment is compared with another active treatment. If sufficient data were available, then separate (or exchangeable) effect modification coefficients could have been estimated for each disease area. However, in our data set, there were 5 or fewer observations of active versus inactive comparisons for all disease areas except oncology; as such, we assumed that the effect modification coefficients were common across disease areas, and this implies that the estimate of B was primarily based on the evidence in oncology. A noninformative prior was assumed for B , that is, $B\;\sim \;N\;(0,\;1000^{2})$ . As for model 1, 3 specifications were tested for π(.) , resulting in models 2a, 2b, and 2c.

Model 2 allowed us to derive more tailored predictive effects in a new RCT conditional on both the disease area and the comparison type:

$$Active\;vs\;Inactive\;comparisons:\;\theta_{pred}\sim N\;\left(\mu_{A^{*}}\;,\;\tau_{A^{*}}^{2}\right)$$

$$Active\;vs\;Active\;comparisons:\;\theta_{pred}\sim N\;\left(\mu_{A^{*}}\;+\;B,\;\tau_{A^{*}}^{2}\right)$$

For disease areas not represented in our data set, 3 levels of predictions were again required:

$$\mu_{pred}\;\sim \;N\;(M,\;\eta^{2})$$

$$\tau_{pred}^{2\;}\;\sim \;\pi \;(\kappa ,\;\nu )$$

$$Active\;vs\;Inactive\;comparisons:\;\theta_{pred}\sim N\;\left(\mu_{pred},\;\tau_{pred}^{2}\right)$$

$$Active\;vs\;Active\;comparisons:\;\theta_{pred}\sim N\;\left(\mu_{pred}+B,\;\tau_{pred}^{2}\right)$$

## Data Sets for Constructing Priors

### Desirable Attributes of a Data Set and Domain of Applicability

The quality and domain of applicability of a set of meta-epidemiological priors will depend on the quality and nature of the data used to construct them. This can be assessed using the criteria below, which are listed in no particular order.

Similarity: the studies included in the data set should be similar to the studies for which priors are required, in the sense that the RTEs can be considered to have been drawn from a common distribution (i.e., exchangeability).^4^ The more similar the studies in the dataset are to the trials of interest, the more reasonable the exchangeability assumption. This aspect covers a number of potentially overlapping criteria a selection of which are discussed here: disease area, outcomes may systematically differ across disease areas; date of publication, trials which are more recent will better reflect contemporary trial proposals; type of interventions, e.g. trials of pharmaceutical interventions will be more similar to those of complex interventions; funding source, priors for publicly funded studies should ideally be derived from publicly funded trials; country, results within countries may be more similar and; trial design, aspects such as comparator and sample size should be similar in the dataset and the contemporary trial proposal.

The number of studies in the data set is another consideration. All else equal, a larger number of trials included in the data set will facilitate more precise estimation of parameters, which will allow for more accurate prior distributions with less uncertainty. Relatedly, larger samples will support fitting a larger range of models.

Minimizing the degree of bias in the included studies is important to producing reliable results. This may be publication bias, which arises when studies with a positive outcome are more likely to be published than those that are less positive.^16^ It may also be bias arising from other issues with study size, design, or execution. Bias adjustment methods may be useful in accounting for this.^17,18^

A rich set of RCT characteristics (i.e., covariates) should be recorded. It is a necessity that there is information on which treatment is the comparator, which is the experimental treatment, and the interpretation of the treatment effect (is a larger value an improvement or deterioration?). A large set of characteristics will allow for more complex meta-epidemiological models, which can produce priors more tailored to specific contexts. This may include information on the treatments (e.g., treatment class), the participants (e.g., average age), and/or the trial design (e.g., number of trial arms). In principle, controlling for these trial-level covariates may be used to mitigate data set limitations, as defined in the “similarity” criterion described above.

### Illustrative Data Set

This article demonstrates meta-epidemiological methods using a data set resulting from a Cochrane review constructed and shared by Djulbegovic et al.^15^ This review was carried out to assess the effectiveness of new treatments compared with established treatments. To minimize publication bias, the authors searched for uninterrupted series of RCTs, which were registered before or at the beginning of the study. Studies were included regardless of publication status. Full information on review methodology and selection criteria is provided in the article.^15^ The data set met the necessary criterion in that it compared new treatments against standard treatments, with a decrease in outcome defined as an improvement.

The authors identified 4 cohorts of studies, 2 of which were in oncology,^19,20^ 1 in neurology,^21^ and 1 in an assortment of diseases.^22^ The data set included 743 RCTs, published between 1955 and 2009, involving a total of 297,744 patients. Some trials assessed more than 2 treatments in separate arms, resulting overall in 877 primary outcome data points. To maximize sample size and following the original analysis by Djulbegovic et al.,^15^ our analysis pooled log odds ratios and log hazard ratios (a sensitivity analysis investigated the impact of this; see Appendix 7). Continuous outcomes were excluded due to low numbers of observations. Table 1 shows the distribution of data points across disease areas. Outcomes pertained to a range of disease areas although were predominately from oncology (87.6%). It was decided that disease areas with fewer than 5 observations should be removed from the data set. This cutoff was chosen because 5 is a recognized rule of thumb to estimate between-trial heterogeneity in conventional meta-analysis.^23^ After removing observations that did not have data on disease area, 828 data points remained. Table 2 assess this data set against the desirable attributes listed above, describing its strengths and limitations.

**Table 1:** Description of the Illustrative Data Set Used to Construct Meta-epidemiology Priors

	*n*	%
Total number of comparisons	828	
Types of comparator in oncology		
Placebo or no treatment	131	15.8
Active	697	84.2
Disease areas		
Circulatory system	22	2.7
Digestive system	5	0.6
Musculoskeletal system	9	1.1
Nervous system	19	2.3
Health status and contact with health services	12	1.4
Injury, poisoning or other external cause	6	0.7
Mental health and behavioral	16	1.9
Obstetrics and gynecology	14	1.7
Oncology	725	87.6

**Table 2:** Quality Assessment of the Illustrative Data Set Used to Construct Meta-epidemiology Priors and Their Domain of Applicability

Criteria	Djulbegovic Data Set
Number of studies	Total number of data points (*n* = 828) is medium in size; however, 87.6% of these are in oncology, and so estimation outside of oncology may be imprecise.
Bias	Publication bias should be low, as the review informing the data set was designed to minimize publication bias. Sets of consecutively conducted RCTs were analyzed regardless of publication status. Other forms of bias were not formally assessed.
Similarity	Disease area: the data set is heterogeneous in disease area, which means modeling is required to adjust for this. Date of publication: publication dates ranged from 1955 to 2010, meaning their relevance to contemporary trials is unclear. Type of interventions: there was a broad range of intervention types, including service delivery, pharmaceuticals, surgery, psychological therapy, and complementary therapy. Funding source: all studies were publicly funded RCTs, which defines the domain of applicability of the resulting priors. Country: all studies were carried out in either the United States or United Kingdom. Trial design: primarily 2-arm RCTs, with a mix of active and passive comparators and a broad range of sample sizes.
Trial characteristics	A broad set of trial characteristics was recorded covering type of publication, disease area, comparator type, sample size. However, no information on intervention type or treatment classes was captured.

RCT, randomized controlled trial.

### Implementation of Statistical Model

All synthesis models were implemented in WinBUGS.^24^ All models were run using 3 Markov chain Monte Carlo chains with different starting values and compared based on residual deviance and the deviance information criterion (DIC). Estimates were obtained from 150,000 iterations (following 50,000 burn-in iterations). Convergence was checked using the Gelman-Rubin diagnostic and visually by assessing the history, chains, and autocorrelation.

## Results

### Estimated Priors for Research Prioritization

All models gave a similar fit to our illustrative data set according to DIC values (see Appendix Table 1). Also, most models resulted in very similar estimates for the predictive distributions (see Appendix Tables 2–4). Although the models that accounted for comparison type (models 2a, 2b, and 2c) may provide more tailored predictive distributions, we highlight that their effect modification coefficient was predominantly based on the available evidence on the oncology disease area. Hence, the estimated covariate effect ( $\bar{x}$ = 0.035, *SE* = 0.032) may not be representative of other disease areas.

**Figure 1:**
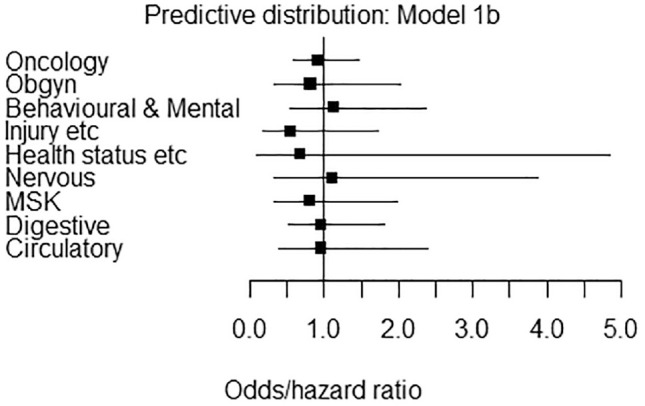
Priors for the relative effect in a future randomized controlled trial according to disease area and comparison type. All estimates are from model 1b and are on the odds/hazard ratio scale.

Table 3 presents the predictive distributions produced by the model that resulted in the lowest DIC (i.e., model 1b). To provide more tailored predictive distributions, we also report separate predictive distributions for active versus active and active versus inactive comparisons only for oncology (this is based on model 2b). The results in Table 3 may be interpreted and operationalized as informative priors by plugging in the appropriate mean and standard deviation into a log-normal distribution. These results are illustrated on the odds/hazard ratio scale in Figure 1.

**Table 3:** Priors for the Relative Effect in a Future Randomized Controlled Trial according to Disease Area and Comparison Type^a^

Disease Area	Predictive Distribution		
	$\bar{x}$	*s*	95% Predictive Interval
1.Circulatory system	−0.046	0.47	−0.967, 0.875
2. Digestive system	−0.033	0.317	−0.654, 0.588
3. Musculoskeletal system	−0.209	0.457	−1.105, 0.687
4. Nervous system	0.108	0.636	−1.139, 1.355
5. Health status and contact with health services	−0.379	0.999	−2.337, 1.579
6. Injury, poisoning, or other external causes	−0.584	0.578	−1.717, 0.549
7. Mental health and behavioral	0.12	0.381	−0.627, 0.867
8. Obstetrics and gynecology	−0.2	0.46	−1.102, 0.702
9.1. Oncology (model 1b)	−0.086	0.24	−0.556, 0.384
9.2. Oncology (model 2b)			
Active v. active comparison	−0.081	0.24	−0.551, 0.389
Active v. inactive comparison	−0.115	0.241	−0.587, 0.357
Other/unknown disease area	−0.147	0.641	−1.403, 1.109

a All estimates are from model 1b, except for oncology, which reports results from both model 1b and 2b. All distributions are reported as normal distributions, (*s*) on the log scale.

Note that the mean of the predictive distribution is negative for all disease areas except “nervous system” and “mental health and behavioral.” A negative predictive mean indicates that new treatments are expected to be more effective than their comparators on average. In this analysis, new nervous system and mental health and behavioral interventions are expected to be less effective than their comparators on average. However, the predictive intervals in all cases span zero, indicating considerable variation in results.

## Case Study: High-Grade Non–muscle-invasive Bladder Cancer

### VOI Methods

Here we demonstrate how the meta-epidemiological priors estimated can be used by analysts to calculate the VOI of funding a bladder cancer trial. We also show how results differ compared with using a “noninformative” prior.

VOI methods use the results of a decision model to calculate the expected health consequences of the uncertainty surrounding a particular parameter or set of parameters.^3^ These health consequences can be converted into monetary terms and used to understand the health benefits that could be gained from commissioning research to resolve parameter uncertainty. Expected value of sample information (EVSI) is a VOI method in which the monetary value of further research is estimated taking account of the study design (including sample size).^6^ As sample size increases, more uncertainty is resolved, resulting in a higher monetary value of research.

### Case Study Model

We obtained a decision model previously employed to inform research prioritization in the United States. This model was developed by Carlson et al.^9,10^ according to minimal modeling principles. Briefly, a Markov model was built to model the disease process of patients with high-grade non–muscle-invasive bladder cancer. In this model, patients either remained recurrence free, had a recurrence (failure), or died. Recurrence affected mortality rate, quality of life, and health care costs. The trial planned to compare standard care with standard care in addition to priming with intradermal Bacillus Calmette-Guérin Tokyo strain 100 µL (0.5 mg/mL). The primary outcome was the rate of recurrence in each arm. The inputs to the decision model are summarized in Table 4. Also included are the trial details including sample size, length of follow-up, and the time during which the information is expected to be valuable.

**Table 4:** Inputs Used in the Bladder Cancer Markov Model and EVSI Calculation Reported in Carlson et al.^10^

Parameter	Value	Source
	(SE or 95% CI)	
Trial inputs		
Sample size of trial	616	Proposal
Average age of the eligible population	68	Proposal
Length of accrual	3 y	Proposal
Length of follow up	2.5 y	Proposal
Decision model inputs		
Expected 1-y failure-free survival rate for the control arm (exponential model)	0.75	Proposal
Overall 5-y disease-specific survival after treatment failure	79%	Huguet et al.^25^
Additional costs for the experiment arm per year	$147.18	CMS^26,27^
Annual health care (surveillance) costs (failure-free patients)	$5,585 (1,117)	Avritscher et al.^28^
Annual health care costs of treating bladder cancer (recurrent patients)	$17,727 (3,545)	Avritscher et al.^28^
Health-related quality of life, failure-free patients	0.997 (0.05)	Kulkarni et al.^29^
Health-related quality of life, recurrent patients	0.96 (0.192)	Kulkarni et al.^29^
Willingness to pay for 1 quality-adjusted life-year	$150,000	Proposal
Population projection inputs		
Size of patient population	25,900	Proposal
Time horizon for information (i.e., length of time trial results inform treatment decisions)	10 y	Assumption
Discount rate	3%	Assumption

CMS, Center for Medicare & Medicaid Services; EVSI, expected value of sample information; SE, standard error.

The annual probability recurrence in the control arm was 25% and assumed known, but recurrence in the treatment arm was uncertain. The aim of the trial was to resolve this uncertainty; however, there were no previous studies to do this. Two approaches were taken to characterize this uncertainty in the treatment arm. First, a “noninformative” prior was used. This was gamma distributed with a shape and rate of 1. This was considered noninformative, as it results in an approximately uniform distribution when transformed to a transition probability. Second was a meta-epidemiological prior from Table 3. The most appropriate estimated prior for this case study was the oncology active versus inactive prior (model 2b). This implies a normally distributed prior for the log hazard ratio with a mean −0.115 and standard deviation 0.241. This was applied to the baseline rate of recurrence, and then a gamma distribution was fit to this distribution to match the model structure (shape = 17.3, rate = 66.1).

EVSI was estimated using a Monte Carlo method. One thousand outer simulations were used to reflect the parameter space of the prior distributions, and 1,000 inner probabilistic simulations were used to reflect nonlinearities in the decision model. It was assumed that only the rate of recurrence would be updated as a result of the trial; this was done using a conjugate Poisson-gamma pair.

## Results

The noninformative prior resulted in an estimate of $44,230 per person and $3.8 billion at the population level. For the estimated prior, the total value of the proposed research was estimated to be $24,109 per person and $2.1 billion for the population, almost half the noninformative estimate.

### Combining Meta-epidemiological Priors with Other Methods

There are approaches other than meta-epidemiology to inform an appropriate prior in the absence of sufficient data, namely, structured expert elicitation and sharing information from indirectly related evidence. These methods are not mutually exclusive, these other methods can improve meta-epidemiological analysis, and vice versa.

Perhaps the most appropriate approach to combining expert elicitation and meta-epidemiology is to integrate meta-epidemiological evidence into expert elicitation exercises. For example, experts may be shown meta-epidemiological evidence to help them form their judgements. This approach is in keeping with the principle of providing experts with all relevant background information.^12^

An alternative approach is to quantitatively combine the results from each method. If it is possible to consider expert elicitation priors and meta-epidemiological priors as independent sources of information, then they may be combined using classical Bayesian updating.^4,30^ However, there is a risk of double counting if the experts considered studies in meta-epidemiology database when forming their judgments.

Another quantitative approach is to use pooling methods. These are common when aggregating the opinions of multiple experts.^31^ It allows for quantitatively combining expert opinions without increasing certainty as more experts are included. In this case, the meta-epidemiological prior is treated as if it is another expert, which may be weighted to increase or decrease its influence on the overall pooled judgement. A limitation of this approach is that the weighting chosen is somewhat arbitrary.

When combining priors from indirect information-sharing methods and meta-epidemiology, classical Bayesian updating may be the most appropriate approach on the condition that the data sets used do not contain common studies.

## Discussion

VOI can be used to make research prioritization more transparent and accountable.^5,32^ At the time of research prioritization decisions, there will be few if any previous studies that can be analyzed to inform an appropriate RTE prior. Therefore, if VOI is to be used routinely by research prioritization bodies to make decisions, it will be necessary to make some judgment about RTE priors in the absence of directly relevant evidence. In this article, we outline a novel method to help inform these judgments. This is the meta-epidemiological framework, which combines the results of RCTs from a variety of disease areas to form predictive distributions that can be used as priors, adjusting for different study characteristics. We have outlined how these priors may be integrated with expert elicitation and methods based on sharing indirect evidence.

We have illustrated this methodology using a data set of 743 trials and synthesized their outcomes with hierarchical models that borrowed strength across disease areas.^15^ This illustrative data set had a number of limitations that limits the validity of our results (these are summarized in Table 2).

There was only a small number of observations in some disease areas. This was partly addressed by using a hierarchical model that shared information across all disease areas.^30,33^ This hierarchal model also enabled us to generate predictions in new disease areas where there was no prior evidence. As larger databases are developed, this step may not be necessary.

There was also limited study-level information included in the data set, and where this information existed, there was limited variation in some disease areas. Priors could not be tailored further than by disease area, and the difference between active versus inactive comparators was estimated using primarily evidence from trials in oncology.

Following Djulbegovic et al., log odds and log hazard ratios were pooled to avoid limiting sample size. The justification for this is that the range of RTEs for these outcomes tend to be similar. We explored this in a sensitivity analysis and found that survival outcomes may demonstrate, on average, lower RTEs than studies reporting binary outcomes (see Appendix 7).

To further tailor the analysis, it may be important to include other covariates that capture differences in mechanism of action, treatment class, and outcome type.^23,34^ Finally, the most recent observations in the data set were from 2009. If the true effects in clinical trials are expected to have changed over time, then this is a challenge to the exchangeability assumption on which this analysis is based. Although there was no statistical evidence of a linear time trend in the data (see Appendix Figure 1), this does not exclude the possibility of more recent or nonlinear changes.

There may also be an important role for using meta-epidemiological priors on their own (“off the shelf”), where there is no other information available and there is insufficient resource for an expert elicitation exercise.^4,9,10,23^ Although we argue that the estimated priors reported here are superior to noninformative priors, due to limitations in the data set, caution is required when applying them in their current form. There is inherent uncertainty involved when making judgments about RTE priors in the absence of previous studies. Because this is inevitable when using VOI in research prioritization, best practice may be sensitivity analysis, in which priors are based on the widest possible range of methods: meta-epidemiology, noninformative prior, expert elicitation, and indirect evidence.

To remedy the above limitations, larger, richer, more up-to-date data sets that cover a wider range of disease areas should be constructed. This would greatly improve the scope and quality of the results. With richer data, more complex models considering additional features of the data will be possible. Then, model 2, which currently includes only 1 covariate that describes whether the RCTs compare 2 active treatments or an active and an inactive treatment, could be extended to include more covariates that further tailor the resulting informative prior to the analysis at hand. For instance, the inclusion of risk of bias indicators as additional covariates, in a similar fashion to that described by Welton et al.,^35^ could potentially alleviate bias considerations and ensure that the derived priors are as valid as possible. These models will be more complex that those reported here, but the fundamental methodology will be that established in this article. It is expected that these models could facilitate the derivation of more tailored informative priors.

In all applications, the limitation of the data set and the domain of applicability of the priors should be considered. Table 2 provides an assessment of the priors reported here and provides a template for reporting in future studies.

The findings in this article are consistent with the published analysis from Djulbegovic et al.,^15^ in which new treatments were associated with improved outcomes on average. However, as shown in Table 3, the priors estimated across disease areas differed in both their means and standard deviations. Notably, the predictive mean for nervous system, mental health, and behavioral disorders was positive. This suggested that new treatments in these disease areas were expected to be slightly worse on average than their comparators. This reflects the poor outcomes with new treatments in these disease areas observed in our data set.

We found that variability in outcomes was larger in certain disease areas, resulting in larger standard deviations and more diffuse predictive distributions. The differences in standard deviations across disease areas observed in our data set were due to differences in heterogeneity of true underlying effects and/or less evidence due to smaller studies used in some disease areas. There may also have been other aspects of trial design or conduct that differed systematically across disease areas that were not captured in our analysis.

The case study compared the differences in the value of additional research resulting from the empirically derived prior and a noninformative prior. The empirical prior provided an estimation of research value that was almost half that of the noninformative prior ($2.1 billion v. $3.8 billion). This was because the plausible range of treatment effects was constrained by the empirical prior. The noninformative prior considered all points between 0% and 100% equally for yearly transition probability for recurrence in the treatment arm. This compares to 25% probability in the control arm. This does not represent a reasonable prior as it overstates uncertainty. Consequently, any analysis based on this prior will overstate the value of further research. By contrast, the empirical prior had a distribution for recurrence rate of 22% (95% interval from 15% to 33%). In the absence of any other evidence, this estimate has greater face validity.

## Conclusion

This work can help to increase the applicability and reliability of Bayesian methods in research design and prioritization by outlining and demonstrating a novel methodology to derive informative priors from data sets of RCT results. Future research is required to improve the validity of these priors by compiling data sets composed of large numbers of up-to-date, low-bias studies, across a wide range of disease areas with a rich set of intervention and study covariates.

## Supplemental Material

sj-docx-1-mdm-10.1177_0272989X231165985 – Supplemental material for Constructing Relative Effect Priors for Research Prioritization and Trial Design: A Meta-epidemiological Analysis Click here for additional data file. Supplemental material, sj-docx-1-mdm-10.1177_0272989X231165985 for Constructing Relative Effect Priors for Research Prioritization and Trial Design: A Meta-epidemiological Analysis by David Glynn, Georgios Nikolaidis, Dina Jankovic and Nicky J. Welton in Medical Decision Making
