# Supplementary material for: Constructing Relative Effect Priors for Research Prioritization and Trial Design: A Meta-epidemiological Analysis
Source: Med Decis Making. 2023 Apr 14;43(5):553–63. doi: 10.1177/0272989X231165985 (PMC10336712; doi:10.1177/0272989X231165985)
Supplement: sj-docx-1-mdm-10.1177_0272989X231165985 – Supplemental material for Constructing Relative Effect Priors for Research Prioritization and Trial Design: A Meta-epidemiological Analysis [file sj-docx-1-mdm-10.1177_0272989X231165985.docx]

# Appendices

## 1.Methods to inform relative effect priors in the absence of direct evidence

Here we outline three methods to inform RTE priors in the absence of direct evidence. We first consider them separately and highlight the limitations of the more common methods (structured expert elicitation and indirect information sharing). However, as described in the section “combining meta-epidemiological evidence with other methods” these methods are not mutually exclusive. For a given context, the most appropriate approach is likely to involve combining two or all three methods so that method-specific limitations can be ameliorated.

### Structured expert elicitation

This is a process used to formally capture the beliefs of individuals identified as experts in a particular area [1]. Elicited beliefs can be captured in form of probability distributions which can be used as priors when empirical data is not available or sparse. Experts’ beliefs about the plausible RTEs have been used when the existing evidence is partial or absent, for example, for sample size calculations or extrapolation [2, 3]. However, planning and conducting elicitation can be resource intensive, experts involved may be subject to motivational bias, and experts’ ability to accurately assess uncertainty is unclear [4, 5].

### Indirect information-sharing

This is the process of combining evidence relating both directly (on the exact population and treatments of interest) and indirectly (on related populations or treatments) to a research question to predict outcomes in the context of interest [6]. In practice, information-sharing is primarily used only when the direct evidence is sparse and excessively uncertain. For example, the U.S. Food and Drug Administration (FDA) and European Medicines Agency (EMA) suggest evidence from adults can be considered for decision-making for paediatric indications. This is due to the regulatory restrictions on trials which hinder evidence development in children [7, 8]. However, this approach requires assumptions that are difficult to verify, and may require highly trained analysts and considerable time.

### Meta-epidemiology

This is an area of research in which large databases of study results are analysed to answer specific epidemiological questions such as estimating the bias due to methodological flaws in RCTs and/or industry sponsorship [9-11]. We are aware of only one meta-epidemiological study to estimate RTEs [12]. Djulbegovic et al. found that new treatments were superior to current treatments on the primary outcome in approximately 60% of trials in a collection of RCTs across a range of disease areas [12]. In addition, the authors reported that 25% of trials report a statistically significant positive result. The results from Djulbegovic et al. were used by Bennette et al. to aid research prioritization in a US oncology setting [13]. For each of the nine research proposals considered for funding there were no previous studies which could be used to construct a reasonable prior for relative effects. The authors constructed a study specific prior for each of the RTEs using the results from Djulbegovic et al. Benette and colleagues assumed that the historical probability of observing a statistically significant positive result (25%) is the same as the probability of achieving the alternative hypothesis in a given trial. By fitting a gamma distribution with 60% density above current practice and 25% density above the alternative hypothesis (reported in sample size calculations) the authors were able to infer a prior for the hazard ratio. Whilst this approach represents a pragmatic response to the challenge of forming relative effect priors, an important limitation was the assumption that the probability of observing a statistically significant positive result is the same as the probability of achieving the alternative hypothesis. Further, this approach is not explicitly grounded in statistical theory which obscures implicit assumptions and limits the possibilities for further extensions and refinements.

## 2.Model fit statistics

Appendix Table 1: Estimated number of parameters, total residual deviance, and DIC across models.

| Model | pD | Total residual deviance | DIC |
| --- | --- | --- | --- |
| Model 1a: Sharing on d and tau. Lognormal on tau | 497.9 | 989.4 | 290.39 |
| Model 2b: Sharing on d and tau. Gamma on tau | 499.16 | 987.8 | 290.16 |
| Model 1c: Sharing on d and tau. Half normal on tau | 499.5 | 986.7 | 290.2 |
| Model 2a: Sharing on d and tau. Lognormal on tau. Active treatment covariate | 498.04 | 990.4 | 292.03 |
| Model 2b: Sharing on d and tau. Gamma on tau. Active treatment covariate | 498.80 | 989.0 | 291.46 |
| Model 2c: Sharing on d and tau. Half normal on tau. Active treatment covariate | 500.02 | 988.2 | 291.21 |

d = disease area specific mean; tau = disease area specific between study heterogeneity; pD = estimated number of parameters; DIC = deviance information criterion

## 3.Predictive distributions for each disease area

Appendix Table 2: Predictive distributions obtained for the true relative effect in a future RCT according to disease area and comparison type. Mean (standard deviation).

| Disease area | N (%) | Model 1a | Model 1b | Model 1c | Model 2a | Model 2b | Model 2c |
| --- | --- | --- | --- | --- | --- | --- | --- |
| Circulatory system | 22  (2.7%) | -0.047 (0.46) | -0.046 (0.47) | -0.046 (0.505) | AA: -0.042 (0.459) | AA: -0.043 (0.47) | AA: -0.043 (0.504) |
|  |  |  |  |  | AI: -0.076 (0.461) | AI: -0.079 (0.471) | AI: -0.078 (0.505) |
| Digestive system | 5  (0.6%) | -0.027 (0.27) | -0.033 (0.317) | -0.035 (0.338) | AA: -0.019 (0.256) | AA: -0.024 (0.304) | AA: -0.027 (0.318) |
|  |  |  |  |  | AI: -0.052 (0.256) | AI: -0.058 (0.304) | AI: -0.06 (0.32) |
| Musculoskeletal system | 9  (1.1%) | -0.206 (0.436) | -0.209 (0.457) | -0.195 (0.521) | AA: -0.2 (0.432) | AA: -0.208 (0.452) | AA: -0.193 (0.517) |
|  |  |  |  |  | AI: -0.235 (0.433) | AI: -0.243 (0.454) | AI: -0.226 (0.518) |
| Nervous system | 19  (2.3%) | 0.103 (0.665) | 0.108 (0.636) | 0.091  (0.691) | AA: 0.111 (0.66) | AA: 0.119 (0.632) | AA: 0.099 (0.689) |
|  |  |  |  |  | AI: 0.074 (0.66) | AI: 0.083 (0.631) | AI: 0.064 (0.69) |
| Health status and contact with health services | 12  (1.4%) | -0.321 (1.25) | -0.379 (0.999) | -0.345  (0.99) | AA: -0.311 (1.24) | AA: -0.374 (0.99) | AA: -0.338 (0.979) |
|  |  |  |  |  | AI: -0.344 (1.239) | AI: -0.407 (0.99) | AI: -0.373 (0.978) |
| Injury, poisoning or other external causes | 6  (0.7%) | -0.566 (0.615) | -0.584 (0.578) | -0.494 (0.688) | AA: -0.564 (0.617) | AA: -0.588 (0.575) | AA: -0.495 (0.685) |
|  |  |  |  |  | AI: -0.598 (0.616) | AI: -0.623 (0.575) | AI: -0.528 (0.686) |
| Mental and behavioural | 16  (1.9%) | 0.121 (0.364) | 0.12 (0.381) | 0.107  (0.404) | AA: 0.121 (0.364) | AA: 0.121 (0.381) | AA: 0.11 (0.403) |
|  |  |  |  |  | AI: 0.086 (0.366) | AI: 0.085 (0.381) | AI: 0.073 (0.404) |
| Obstetrics and gynaecology | 14  (1.7%) | -0.197 (0.447) | -0.2  (0.46) | -0.185 (0.501) | AA: -0.196 (0.448) | AA: -0.199 (0.459) | AA: -0.186 (0.501) |
|  |  |  |  |  | AI: -0.229 (0.45) | AI: -0.236 (0.46) | AI: -0.221 (0.504) |
| Oncology | 725 (87.6%) | -0.086 (0.239) | -0.086 (0.24) | -0.086 (0.239) | AA: -0.08 (0.239) | AA: -0.081 (0.24) | AA: -0.08 (0.239) |
|  |  |  |  |  | AI: -0.114 (0.24) | AI: -0.115 (0.241) | AI: -0.115 (0.241) |

AA : Active vs active comparison, AI: Active vs Inactive comparison, N: number of studies included in disease area, RCT : randomised controlled trial.

All distributions reported as Normal distributions mean (standard deviation) on the log scale

## 4.Predictive distribution in unknown disease area

Appendix Table 3: Predictive distributions for the true effect obtained for the true relative effect in a future RCT in an unknown disease area. Mean (standard deviation).

| Model | Mean (standard deviation) |
| --- | --- |
| Model 1a | -0.135 (1.005) |
| Model 1b | -0.147 (0.641) |
| Model 1c | -0.132 (0.658) |
| Model 2a | AA: -0.132 (1.026) |
|  | AI: -0.168 (1.025) |
| Model 2b | AA: -0.142 (0.638) |
|  | AI: -0.177 (0.64) |
| Model 2c | AA: -0.129 (0.655) |
|  | AI: -0.164 (0.656) |

AA: Active vs active comparison, AI: Active vs Inactive comparison

All distributions reported as Normal distributions mean (standard deviation) on the log scale

## 5.Estimates of between studies standard deviation

Appendix Table 4: Estimated between-studies standard deviation across models. Posterior mean (standard deviation).

| Disease area | N (%) | Model 1a | Model 1b | Model 1c | Model 2a | Model 2b | Model 2c |
| --- | --- | --- | --- | --- | --- | --- | --- |
| Circulatory system | 22  (2.7%) | 0.45 (0.08) | 0.46 (0.08) | 0.49 (0.1) | 0.45 (0.08) | 0.46 (0.08) | 0.49 (0.1) |
| Digestive system | 5  (0.6%) | 0.25 (0.08) | 0.3  (0.09) | 0.32 (0.13) | 0.24 (0.07) | 0.28 (0.08) | 0.3 (0.12) |
| Musculoskeletal system | 9  (1.1%) | 0.42 (0.12) | 0.44 (0.11) | 0.5 (0.16) | 0.41 (0.12) | 0.43 (0.11) | 0.5 (0.16) |
| Nervous system | 19  (2.3%) | 0.65 (0.19) | 0.62 (0.16) | 0.68 (0.18) | 0.64 (0.19) | 0.61 (0.16) | 0.67 (0.18) |
| Health status and contact with health services | 12  (1.4%) | 1.22  (0.7) | 0.97 (0.35) | 0.96 (0.31) | 1.21 (0.68) | 0.96 (0.34) | 0.95 (0.3) |
| Injury, poisoning or other external causes | 6  (0.7%) | 0.55 (0.35) | 0.52  (0.2) | 0.63 (0.26) | 0.55 (0.33) | 0.52  (0.2) | 0.62 (0.26) |
| Mental and behavioural | 16  (1.9%) | 0.35 (0.06) | 0.37 (0.06) | 0.39 (0.08) | 0.35 (0.06) | 0.37 (0.06) | 0.39 (0.07) |
| Obstetrics and gynaecology | 14  (1.7%) | 0.43 (0.09) | 0.44 (0.09) | 0.48 (0.11) | 0.43 (0.09) | 0.44 (0.09) | 0.44 (0.09) |
| Oncology | 725 (87.6%) | 0.24 (0.01) | 0.24 (0.01) | 0.24 (0.01) | 0.24 (0.01) | 0.24 (0.01) | 0.24 (0.01) |

## 6.Observed outcomes over time


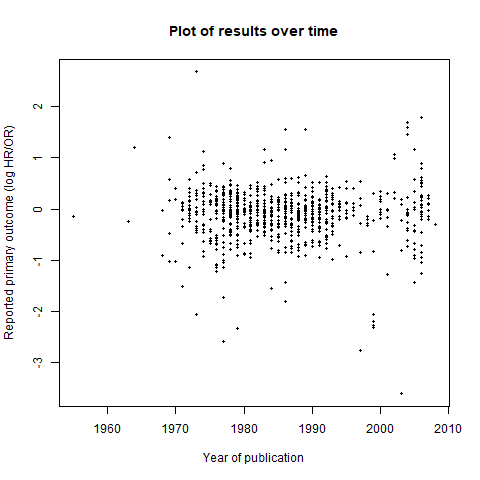


Appendix Figure 2: Plot of observed point estimates in the Djulbegovic dataset by publication year (n = 828)

## 7. Sensitivity analysis

We further explored the impact of the outcome type, publication year, and trial size using a simplified form of Model 2 where the mean and the covariate effects were assumed to be independent of disease area, so that:

$$\theta_{i} \sim N (\mu+\beta X, \tau^{2})$$

where $X$ represents a binary or continuous covariate that is suspected to affect mean effect $\mu$, and $\tau$ represents the between-trials standard deviation. Depending on whether $X$ is a binary or a continuous covariate, $\beta$ represents either the additional effect when $X=1$ or the additional effect per unit of $X$, respectively. Vague priors were used for the hyperparameters i.e., $\mu\sim N (0, {10}^{-6})$, $\beta\sim N (0, {10}^{-6})$, and $\tau\sim U (0, 5)$.

Four scenario analyses were undertaken overall:

1. In scenario 1, we explored whether trials using survival outcomes (and hence reporting relative effects as hazard ratios) demonstrated a different effect than trials using a response rate outcome (and reporting relative effects as odds ratios). When a response rate outcome was used, we assumed $X=1$; hence, $\beta$ represented the average relative effect increment of using a binary outcome instead of a survival outcome. Given that our database comprised studies reporting different kinds of survival outcomes (OS, PFS, EFS), we undertook two different sub scenarios:
   1. In 1a, we analysed 423 studies comprising 310 studies reporting OS/PFS and 113 studies reporting a response rate outcome
   2. In 1b, we enriched the dataset used in 1a and analysed 663 studies comprising 550 studies reporting any survival outcome (310 studies reporting OS/PFS and 240 reporting EFS), and 113 studies reporting a binary outcome
2. In scenario 2, we explored whether an association could be identified between the treatment effect and the trials’ publication year. The publication year covariate $X$ was centered to improve model fit and prevent convergence and autocorrelation issues
3. In scenario 3, we explored whether the trial size, proxied by the standard error of its treatment effect, could influence the size of reported relative effect.

The scenario analyses results are shown below:

Appendix Table 6: scenario analysis results

| Scenario | $\mu$  (95% CrI) | $\beta$  (95% CrI) | $\tau$  (95% CrI) |
| --- | --- | --- | --- |
| Scenario 1 |  | | |
| Scenario 1a | 0.0023  (-0.069, 0.073) | -0.062  (-0.139, 0.013) | 0.177  (0.148, 0.209) |
| Scenario 1b | -0.076*  (-0.100, -0.052) | -0.086*  (-0.162, -0.010) | 0.223  (0.200, 0.247) |
| Scenario 2 | -0.096*  (-0.124, -0.069) | -0.002  (-0.005, 0.001) | 0.337  (0.313, 0.362) |
| Scenario 3 | -0.087*  (-0.141, -0.034) | -0.052  (-0.295, 0.188) | 0.338  (0.348, 0.363) |

The table shows the medians for parameters $\mu$, $\beta$, and $\tau$. * indicates a statistically significant posterior estimate at the 5% significance level.

Overall, the results align with expectations. Scenario 1 analyses resulted in a negative treatment effect modification coefficient, albeit statistically significant only for Scenario 1b, suggesting that studies reporting survival outcomes demonstrate, on average, lower relative treatment effects than studies reporting binary outcomes. This observation could be explained by the fact that survival outcomes are predominantly used in oncology trials which may result in smaller effects than other disease fields. Regarding Scenario 2, the posterior estimates suggest that more recent studies report, on average, smaller relative effects. Despite that this result is borderline statistically insignificant, it is in line with previous work which demonstrated that older studies produced more enthusiastic effects [14]. Finally, the posterior estimates in Scenario 3 suggest that smaller studies report, on average, more exaggerated relative effects than larger studies which aligns with previous findings from selection models in the publication bias literature [15, 16].

## References

[1] O'Hagan A, Buck CE, Daneshkhah A, Eiser JR, Garthwaite PH, Jenkinson DJ, et al. Uncertain judgements: eliciting experts' probabilities: John Wiley & Sons 2006.

[2] Bojke L, Claxton K, Bravo-Vergel Y, Sculpher M, Palmer S, Abrams K. Eliciting distributions to populate decision analytic models. Value in Health. 2010; 13(5):557-64.

[3] Stevenson MD, Oakley JE, Lloyd Jones M, Brennan A, Compston JE, McCloskey EV, et al. The cost-effectiveness of an RCT to establish whether 5 or 10 years of bisphosphonate treatment is the better duration for women with a prior fracture. Medical Decision Making. 2009; 29(6):678-89.

[4] Tversky A, Kahneman D. Judgment under uncertainty: Heuristics and biases. science. 1974; 185(4157):1124-31.

[5] Tversky A, Kahneman D. Belief in the law of small numbers. Psychological bulletin. 1971; 76(2):105.

[6] Spiegelhalter DJ, Best NG. Bayesian approaches to multiple sources of evidence and uncertainty in complex cost‐effectiveness modelling. Statistics in medicine. 2003; 22(23):3687-709.

[7] EMA. Extrapolation of efficacy and safety in paediatric medicine development. 2018.

[8] FDA. Leveraging Existing Clinical Data for Extrapolation to Pediatric Uses of Medical Devices: Center for Devices and Radiological Health; 2016.

[9] Savović J, Turner RM, Mawdsley D, Jones HE, Beynon R, Higgins JP, et al. Association between risk-of-bias assessments and results of randomized trials in Cochrane reviews: the ROBES meta-epidemiologic study. American Journal of Epidemiology. 2018; 187(5):1113-22.

[10] Turner RM, Jackson D, Wei Y, Thompson SG, Higgins JP. Predictive distributions for between-study heterogeneity and simple methods for their application in Bayesian meta-analysis. Stat Med. 2015; 34(6):984-98.

[11] Djulbegovic B, Lacevic M, Cantor A, Fields KK, Bennett CL, Adams JR, et al. The uncertainty principle and industry-sponsored research. The Lancet. 2000; 356(9230):635-8.

[12] Djulbegovic B, Kumar A, Glasziou PP, Perera R, Reljic T, Dent L, et al. New treatments compared to established treatments in randomized trials. Cochrane Database Syst Rev. 2012; 10:MR000024.

[13] Bennette CS, Veenstra DL, Basu A, Baker LH, Ramsey SD, Carlson JJ. Development and Evaluation of an Approach to Using Value of Information Analyses for Real-Time Prioritization Decisions Within SWOG, a Large Cancer Clinical Trials Cooperative Group. Med Decis Making. 2016; 36(5):641-51.

[14] Salanti G, Marinho V, Higgins JP. A case study of multiple-treatments meta-analysis demonstrates that covariates should be considered. J Clin Epidemiol. 2009; 62(8):857-64.

[15] Trinquart L, Chatellier G, Ravaud P. Adjustment for reporting bias in network meta-analysis of antidepressant trials. BMC medical research methodology. 2012; 12(1):1-11.

[16] Mavridis D, Sutton A, Cipriani A, Salanti G. A fully Bayesian application of the Copas selection model for publication bias extended to network meta-analysis. Stat Med. 2013; 32(1):51-66.
